# A bibliometric and visual analysis of Jak1 to explore trends and frontiers

**DOI:** 10.3389/fonc.2025.1537508

**Published:** 2025-07-17

**Authors:** Cong Yu, Jiamin Cao, Xiaodong Xie, Wenguang Chen, Ensi Hong

**Affiliations:** ^1^ Graduate School, Jiangxi University of Traditional Chinese Medicine Nanchang, Jiangxi, China; ^2^ Nanchang Normal University, Infirmary of the Logistics Support Department, Nanchang, Jiangxi, China; ^3^ The Affiliated Hospital of Jiangxi University of Chinese Medicine, Nanchang, China

**Keywords:** jak1, bibliometric, visual analysis, research hotspots, citespace

## Abstract

**Background:**

JAK1, a member of the JAK kinase family, is involved in the signal transduction of multiple cytokine pathways and is crucial in the onset and progression of inflammation and tumours. Consequently, JAK1 has garnered significant attention in recent years.

**Methods:**

We use bibliometric and visual analysis to evaluate the thematic trends and knowledge structure of TRPV1’s research papers on JAK1, sourced from the Web of Science core collection from 2003 to 2024. CiteSpace is used to analyze references and keywords of authors, institutions, countries, and commonly cited, and applies co-current and clustering functions to generate visual knowledge maps.

**Results:**

A total of 3,686 articles were incorporated. The primary research domain of JAK1 is oncology; the United States leads in publication volume, with the University of Texas holding the most prominent central position. The keyword distribution indicates that the literature on JAK1 from 2003 to 2009 primarily concentrated on mechanistic studies, encompassing gene expression, activation, pathways, and cell apoptosis. From 2008 to 2018, research hotspots predominantly examined the association between JAK1 and various disease atlases. Beginning in 2012 and extending to 2024, the focus shifted towards the research and development of clinical pharmaceuticals, along with their safety and efficacy. Gene expression, signal transduction, atopic dermatitis, and JAK1-selective inhibitors have emerged as prominent research areas in recent years, exhibiting significant potential for development.

**Conclusion:**

This study presents the contemporary status and prospective trends of JAK1 research over the last two decades. Current research focuses on skin inflammation, rheumatoid arthritis, and tumor-related diseases, while new signaling pathways are constantly being discovered. JAK1 inhibitors are gradually being used in clinical practice and have good development prospects, which will become the main trend of future research.

## 1 Introduction

Janus kinase (JAK) comprises a family of four enzymes. JAK1, JAK2, JAK3, and tyrosine kinase 2 (TYK2) transmit downstream signals of cytokines via the JAK/STAT pathway, modulate the expression of target genes, and engage in processes such as cell proliferation, differentiation, and immune regulation (1, 2), which is closely related to a variety of diseases (3). The JAK1 subtype is the common core of multiple pathways. Different JAK family members tend to control different STATs. After every 2–3 JAK molecules combine to form a polymer, they are jointly responsible for a pathway, producing specific biological effects. JAK1 is closely related to inflammation, cancer, immunity, and other diseases (4–6), JAK2 is primarily related to diseases of the blood system (7, 8), and JAK3 is related to a variety of autoimmune diseases (9, 10). Among the members of the JAK family, JAK1 is the only subtype that can form heterodimers with all three types of JAK, and JAK1 also participates in the information transduction of various signaling pathways. Currently, JAK1 has emerged as a therapeutic target for numerous immune and inflammatory disorders. Nevertheless, the thematic trends and knowledge structure of JAK1 have not been examined through bibliometric analysis. The bibliometric analysis method can objectively analyze the current situation and development trend of the discipline, devoid of personal subjective judgment, thereby accurately reflecting the present development status, research focal points, and prospective trends (11, 12). Therefore, this study adopts bibliometric analysis to perform a thorough review of JAK1 studies from 2003 to 2024 in order to understand the co-citation of literature, establish a research collaboration network, and evaluate research trends and emerging hotspots.

## 2 Materials and methods

### 2.1 Source of data and search strategy

**Figure 1:**
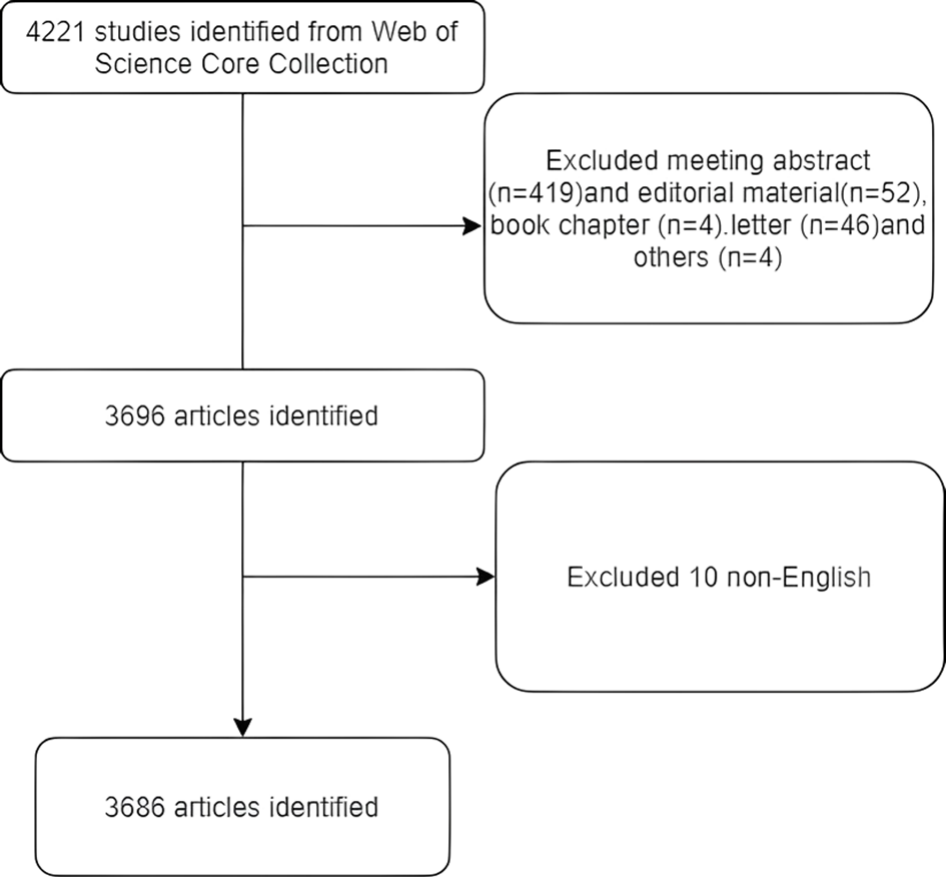
Search flowchart.

The literature retrieval was conducted online via the Web of Science core collection Science Citation Index-Expanded, on September 1, 2024. The data search strategy is as follows: Topic: JAK1; Date: 2003-01–01 to 2024-09-01; Article type: full text; Following the removal of duplicates, a total of 4,221 documents were ultimately incorporated into the literature measurement analysis. Excluding conference papers, editorial materials, book chapters, letters, and other unrelated documents, 3,696 articles remain. Excluding non-English literature, there remain 3,686 articles. **Figure 1** illustrates the search flowchart.

### 2.2 Data analysis

The English literature is exported in”RefWorks” format, and the literature in WOS is exported in”Plain text” format and renamed download * *. txt. After converting data and creating a new project in the CiteSpace software, data analysis is carried out according to the selection. The CiteSpace parameter is as follows: The time span was from January 2003 to September 2024, and the time slices were “1 year per slice.”; Node selection type, one at a time, and other parameters in the configuration function area are set according to the default value of the system. The broader the range of nodes on the map, the higher the frequency (or reference frequency) of the analyzed research object; the color and thickness of the node’s inner circle denote the frequency of occurrence across various time periods; the connecting lines between nodes illustrate the co-occurrence (or co-citation) relationship, with thickness signifying the strength of this co-occurrence (or co-citation), and color indicating the initial time of co-occurrence (or co-citation) of the nodes. The purple circles signify mediating centrality, with nodes exhibiting high centrality deemed more significant (13).

### 2.3 Results

#### 2.3.1 Analysis of publications

**Figure 2:**
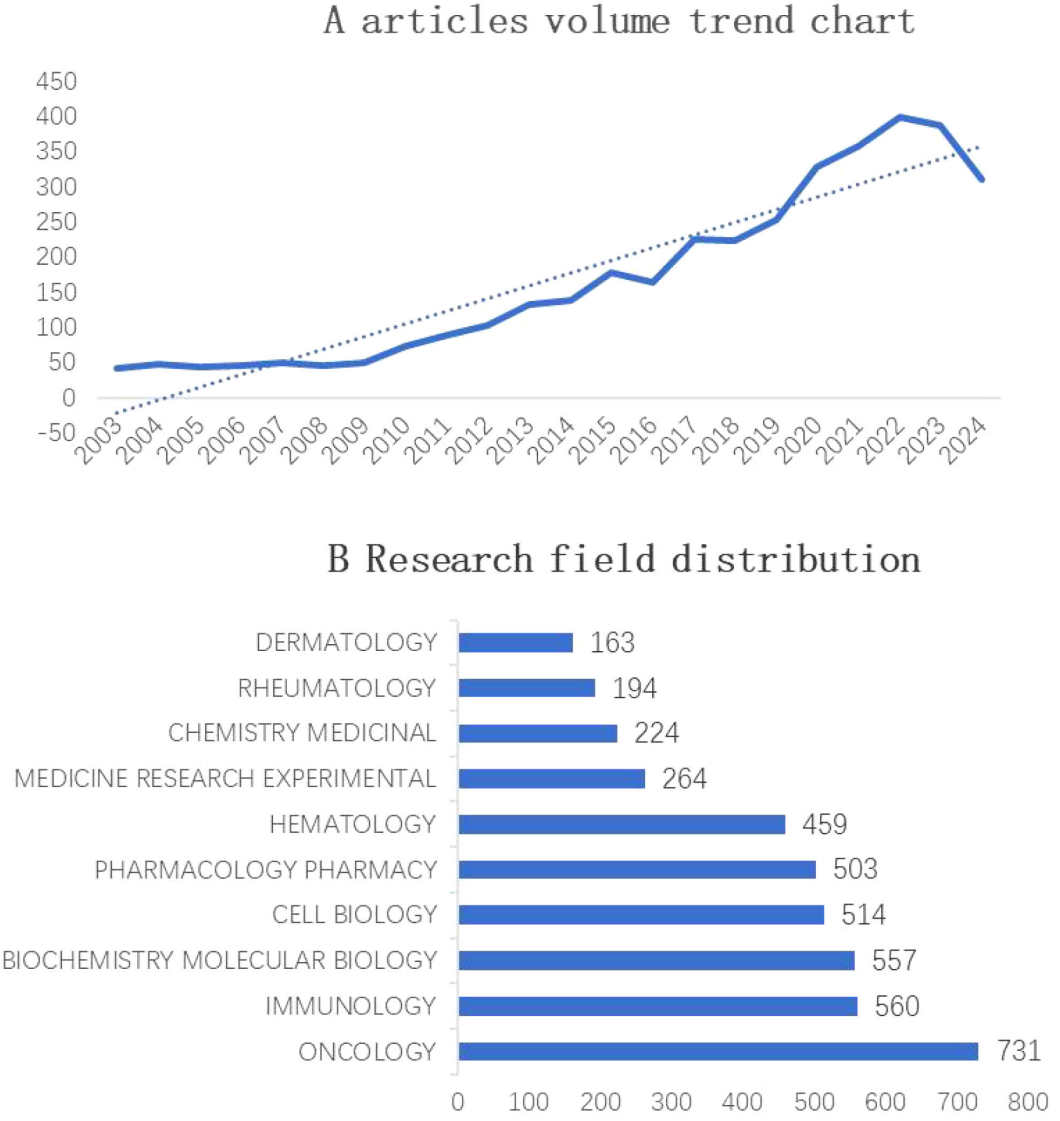
**(A)** Annual publication trends in JAK1 research (2003–2024). X-axis: Year (2003–2024); Y-axis: Number of publications. The dashed line represents a polynomial trendline illustrating three growth phases. **(B)** Top 10 research fields of JAK1 research (2003–2024).

The Web of Science Core Collection included a total of 3,686 articles from 2002 to 2024, with the overall trend categorized into three phases. The initial phase spanned from 2003 to 2010, a stable period, and the annual publication volume remained basically at the same level. The second stage was from 2011 to 2020, a development period, and the overall trend of publication volume continued to rise. After 2020, the growth rate began to increase markedly and peaked in 2022 (399 articles), indicating that JAK1 research continues to attract the attention of researchers and has good development potential. See **Figure 2A** for details.

#### 2.3.2 Analysis of the research field

The top three research domains for JAK1 are oncology (642 articles), biochemistry (541 articles), and immunology (507 articles), collectively accounting for 46% of all JAK1-related publications. **Figure 2B** shows the top 10 research fields of JAK1 from 2002 to 2024: oncology, biochemistry, immunology, cell biology, pharmacology, hematology, medical research, medicine, chemistry, multidisciplinary science, and dermatology.

#### 2.3.3 Analysis of countries/regions

**Figure 3:**
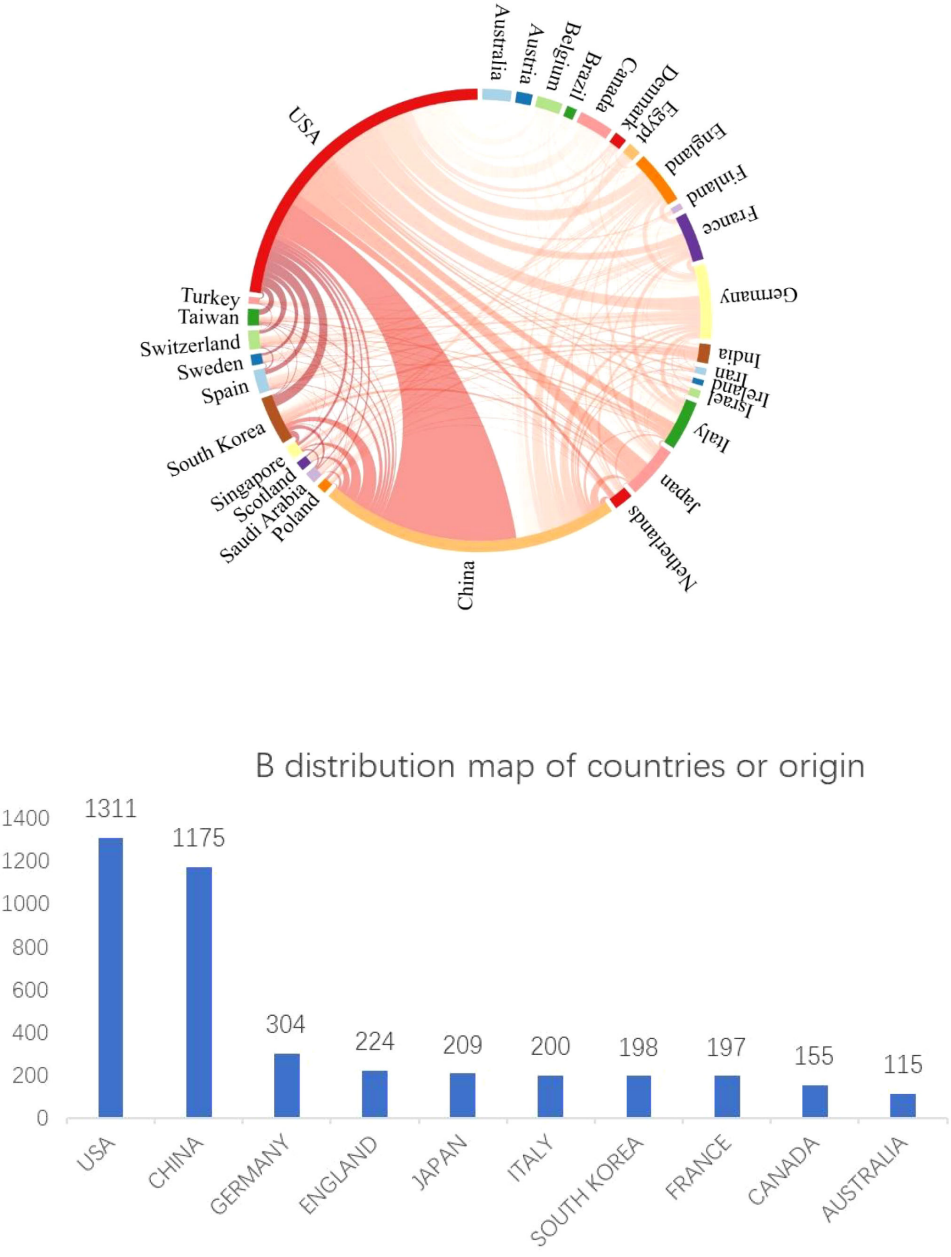
**(A)** Global collaboration network in JAK1 research (Citespace Analysis). **(B)** Top 10 countries by JAK1 publication volume (2003–2024).

In total, 86 countries or regions have disseminated research papers on JAK1. We utilized Citespace software to construct a collaborative network of countries, as depicted in **Figure 3A**. Every node signifies a country. In the cooperation network of the issuing country, a larger node signifies a greater volume of documents dispatched by the country; a denser connection indicates a closer collaboration among entities. The connection line in the network signifies the collaboration between countries, while the line’s thickness denotes the degree of cooperation. **Figure 3A** illustrates that the United States and China are the foremost nations in JAK1 research, signifying their substantial influence in this domain and extensive collaboration with other countries. **Figure 3B** illustrates the top 10 countries by publication volume, which collectively represent 74% of the total publications, highlighting the concentration of nations engaged in JAK1-related research.

#### 2.3.4 Analysis of institutions

**Figure 4:**
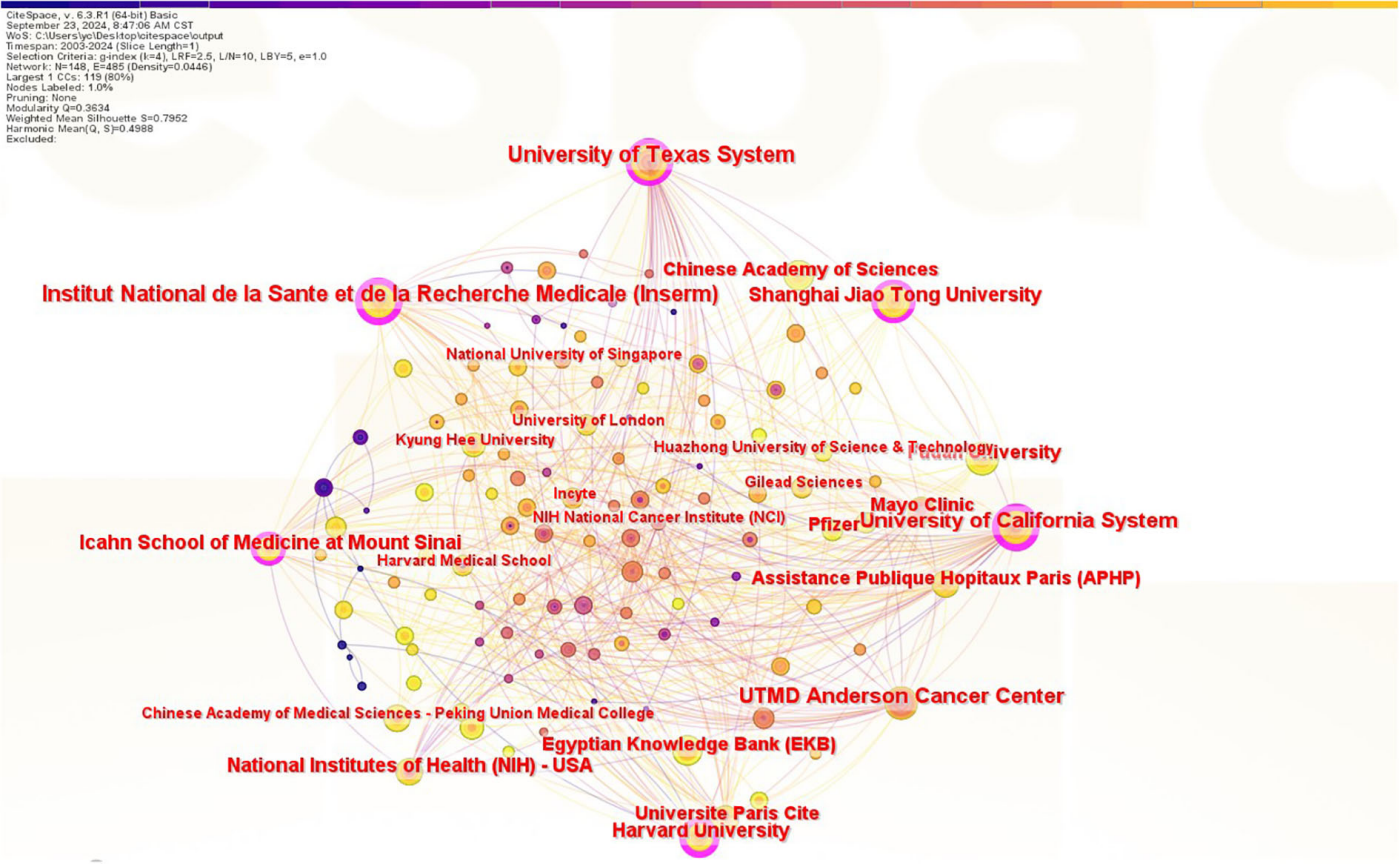
A JAK1 field of issuing organisations cooperation network map (2003–2024).

As shown in **Figure 4**, a total of 199 institutions engaged in JAK1 research, with 6 institutions exhibiting centrality values of ≥0.1, indicating that multiple institutions are publishing key or transformative research. The University of California has the highest centrality (centrality = 0.38) as shown in **Table 1**. The University of Texas has the most publications, followed by the French National Institute of Health and Medical Research, the University of California, the Icahn School of Medicine at Mount Sinai, Harvard University, Shanghai Jiao Tong University, the National Institutes of Health, the Paris Hospital Public Assistance (APHP), and the University of Paris. However, the collaboration between the main publishing institutions with the largest number of publications is weak.

**Table 1:** Top 10 issuing institutions in the field of jak1(2003–2024).

Rank	Institutions	Count/article
1	UniversityofTexasSystem	132
2	InstitutNationaldelaSanteetdelaRechercheMedicale(Inserm)	114
3	UniversityofCaliforniaSystem	110
4	UTMDAndersonCancerCenter	106
5	IcahnSchoolofMedicineatMountSinai	87
6	HarvardUniversity	83
7	ShanghaiJiaoTongUniversity	83
8	NationalInstitutesofHealth(NIH)-USA	73
9	AssistancePubliqueHopitauxParis(APHP)	72
10	UniversiteParisCite	71

#### 2.3.5 Analysis of the author

We identified core authors using Price’s law. The calculation formula is $N=0.749\times \sqrt{\eta_{\max}}$ , where ηmax represents the highest number of publications by an author, and authors with a publication count ≥N are considered core authors. Calculations reveal that the core author’s volume is ≥6 (5.554) articles, totaling 35 authors; The number of articles and the top 10 authors cited are shown in **Table 2A**, including Verstovsek, Srdan (55 articles), Ahn, KwangSeok (21 articles), Tanaka, Yoshiya (14 articles), Heinrich, PC (11 articles), Set Hi, Gautam (10 articles) is the top five authors in the volume of articles. The number of cited articles is the total number of cited articles for all articles published in the JAK1 research area by each author, as shown in **Table 2B**. The top five authors cited are OSHEAJJ (399 TIMES), VERSTOVSEKS (369 TIMES), QUINTÁS-CARDAMAA (234 TIMES), TEFFERIA (228 TIMES), and GHORESCHIK (213 TIMES).

**Table 2A:** Top 10 authors in the field of Jak1 (2003–2024).

Rank	Authors	Published articles/articles
1	Verstovsek,Srdan	55
2	Ahn,KwangSeok	21
3	Tanaka,Yoshiya	14
4	Heinrich, PC	11
5	Sethi, Gautam	10
6	Vannucchi,AlessandroM	9
7	Danese, Silvio	9
8	Mesa, Ruben A	9
9	Chang, Christine	7
10	Johnson, Adam	7

**Table 2B:** Top 10 authors cited in the Jak1 field ranking(2003–2024).

Rank	Authors	Cited times
1	OSHEAJJ	399
2	VERSTOVSEKS	369
3	QUINTÁS-CARDAMAA	234
4	TEFFERIA	228
5	GHORESCHIK	213
6	HARRISONC	206
7	MESARA	176
8	SCHWARTZDM	168
9	YUH	159
10	PARDANANIA	150

#### 2.3.6 Keyword co-occurrence analysis

**Figure 5:**
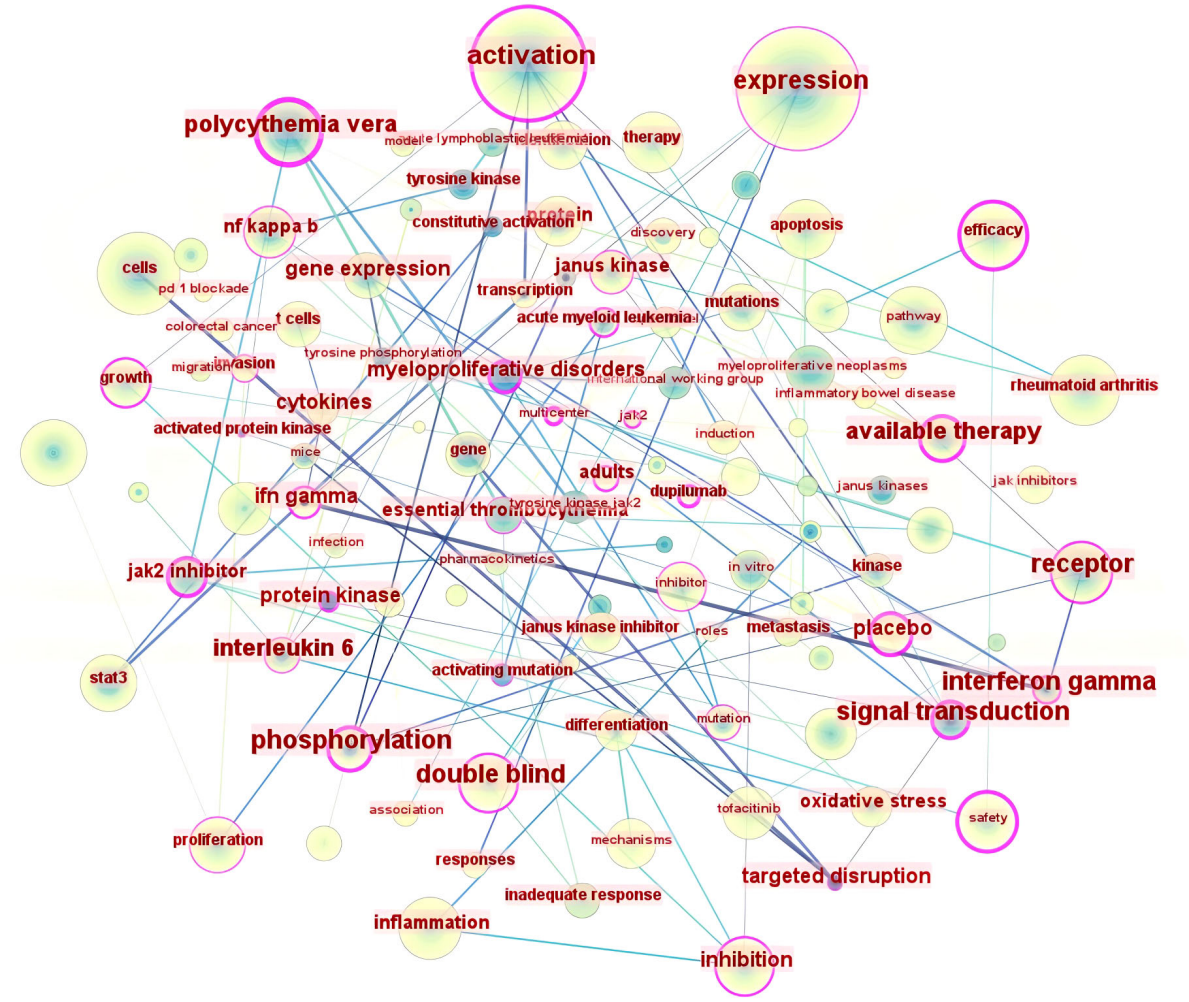
A literature keyword co-occurrence map (2003–2024).

Keywords typically serve to summarize and condense, thereby enhancing comprehension of the article’s principal themes. The literature keyword co-occurrence map (**Figure 5**) comprises 174 nodes and 1461 links. The twenty most frequent keywords (**Table 3**) pertain to mechanisms including gene expression (14), activation (15), pathways (16), and apoptosis (17), and are closely associated with diseases such as rheumatoid arthritis and cancer. They also encompass the analysis of safety and efficacy in clinical treatment. The keywords exhibiting a centrality exceeding 0.1 in the keyword co-occurrence analysis are expression (18), activation (19), cells (20), and rheumatoid arthritis (21), indicating that mechanistic research and rheumatoid arthritis are significant aspects of JAK1 research.

**Table 3:** Top 20 high-frequency keywords in the field of Jak1 (2003–2024).

Keywords	Frequency	Centrality
expression	624	0.15
activation	534	0.14
cells	309	0.16
pathway	215	0.06
rheumatoidarthritis	207	0.1
cancer	203	0.04
receptor	180	0.05
efficacy	176	0.07
inflammation	167	0.04
apoptosis	166	0.05
polycythemiavera	163	0.07
stat3	161	0.07
inhibition	159	0.05
therapy	155	0.07
geneexpression	152	0.05
safety	132	0.04
doubleblind	128	0.07
proliferation	126	0.05
disease	121	0.03
protein	119	0.05

#### 2.3.7 Keyword cluster graph

**Figure 6:**
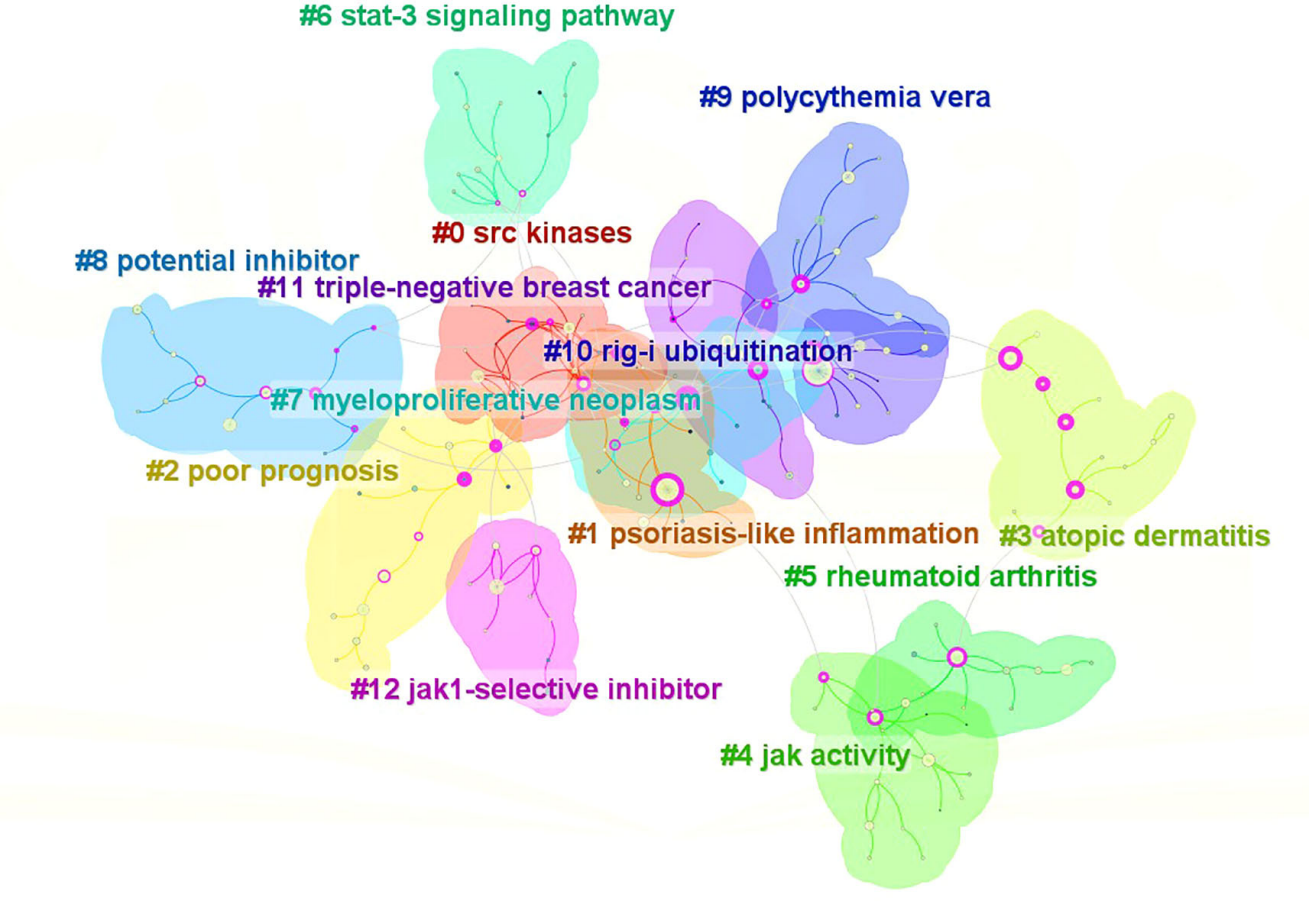
A keyword cluster analysis in the field of Jak1 (2003–2024).

Utilizing the keyword co-occurrence map, the log-likelihood ratio (LLR) algorithm clusters the literature (**Figure 6**), resulting in 13 cluster labels. The cluster labels of the literature keywords are: #0(srckinasesSRC), #1(psoriasis-likeinflammation), #2(poorprognosis), #3(atopicdermatitis), #4(jakactivityjak), #5(rheumatoidarthritis), #6(stat-3signalingpathway), #7(myeloproliferativeneoplasm), #8(potentialinhibitor), #9(polycythemiavera), #10(rig-iubiquitination), #11(triple-negativebreastcancer), #12(jak1-selectiveinhibitor).Clusters #0, #4, #6, and #10 primarily investigate the mechanism of JAK1; clusters #1, #3, #5, #7, #9, and #11 predominantly examine the disease spectrum of JAK1; clusters #8 and #12 chiefly focus on the clinical application of JAK1.

The literature cluster module value (Q)=0.7927(>0.3) signifies that the network structure of the cluster results is exceptional. The average contour value (S) serves as a metric for assessing the homogeneity of the network. A value approaching 1 indicates greater homogeneity within the network. If the value exceeds 0.7, it indicates that the cluster results are reliable. This study reveals that the average outline value for clustering in Chinese and English literature is 0.9374 (>0.7), signifying the credibility of the clustering results. The keyword cluster map of the literature indicates that each cluster overlaps and intersects, demonstrating the strong interconnection among them. The literature primarily focuses on the mechanistic research of JAK1 and the clinical investigation of surgical and tumor-associated diseases linked to skin inflammation.

#### 2.3.8 Keyword timeline chart and keywords with the strongest citation bursts analysis

**Figure 7:**
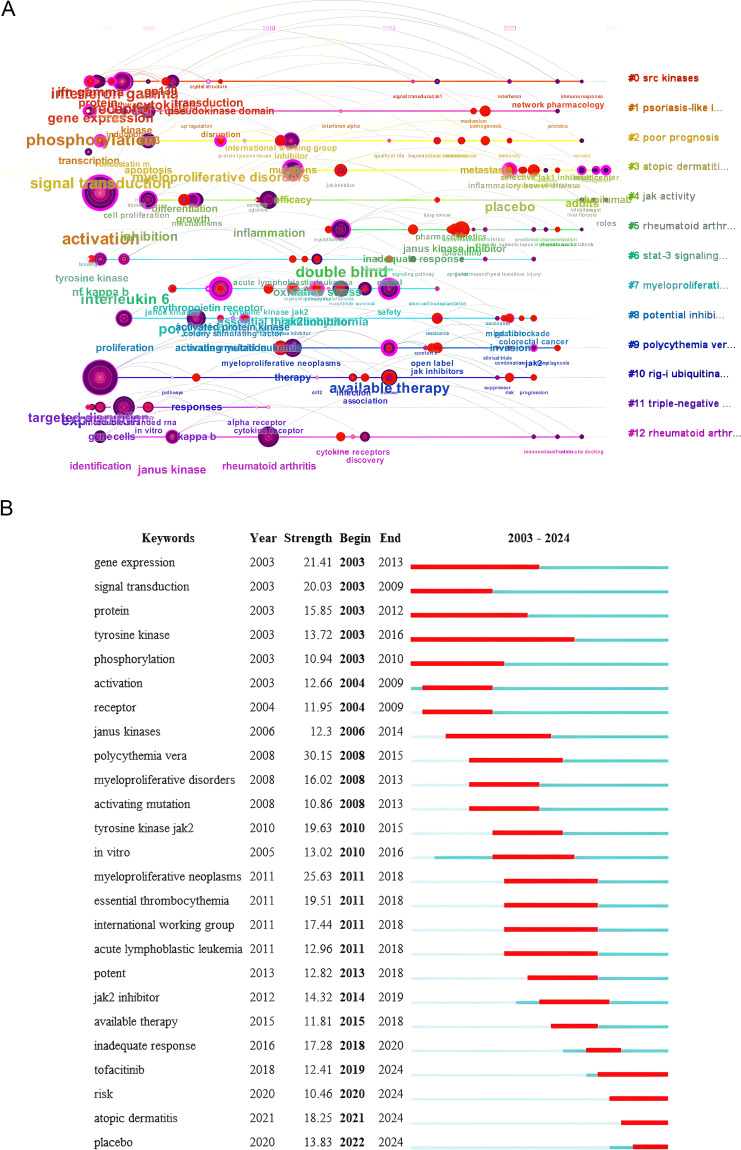
**(A)** Keyword timeline chart (2003–2024). **(B)** Keywords with the strongest citation bursts (2003–2024).

A keyword timeline chart and highlight analysis facilitate comprehension of the research landscape in this domain and enable the identification of research trends. This study employs the keyword cluster map, utilizing the year as the horizontal axis and the cluster label as the vertical axis, to analyze the keyword timeline diagram using CiteSpace software (**Figure 7A**).

The keyword timeline diagram shows that the SRC kinase (cluster #0) has been widely studied since 2003. Atopic dermatitis (cluster #3) and JAK1-selective inhibitors (cluster #12) have become research hotspots in recent years. This implies that the main research focus and trend of JAK1 in the future will focus on clinical trials and mechanism research.

Keywords with the Strongest Citation Bursts refer to a significant increase in the frequency of a certain keyword in a short period, which indicates that research with high attention during this period can be used to determine hot spots and trends in the research field. “Begin” and “End” indicate the start and end time of Keywords with the strongest citation bursts, and “Strength” indicates the strength of Keywords with the strongest citation bursts. Increased strength correlates with enhanced influence.

**Figure 7B** shows the keywords with the strongest citation bursts analysis of JAK1 research literature. The top five keywords with the strongest citation bursts are polycythemiavera, myeloproliferativeneoplasms, geneexpression, signaltransduction, tyrosinekinasejak2. From the perspective of the length of highlight time, JAK1 literature research can be divided into three stages: from 2003 to 2009, the research focus was on the mechanism of JAK1; from 2008 to 2018, the main focus was on the combination of JAK1 and clinical diseases; from 2012 to 2024, the research focus began to shift to the development of JAK1 clinical drugs and their safety and efficacy.

## 3 Discussion

### 3.1 Advancements in understanding the mechanism of action of JAK1-associated signaling pathways

#### 3.1.1 Inhibit the proliferation of cancer cells

Numerous studies have demonstrated that JAK1-related signaling pathways exhibit significant Anti-cancer efficacy (22, 23). The JAK/STAT signaling pathway suppresses the proliferation, differentiation, and apoptosis of tumor cells by inhibiting the activity of the suppressor of cytokine signaling (SOCS) family (24). In patients with colorectal cancer, JAK1 overexpression was significantly correlated with reduced survival, and the inhibition of JAK1 protein expression can impede cancer cell proliferation and progression, thereby effectively treating tumors (25). Moreover, JAK/STAT signaling pathways can combat cancer by modulating energy metabolism (26), influencing the immune microenvironment (27), and managing drug resistance (28), among other mechanisms.

#### 3.1.2 Regulate cell autophagy

Autophagy is the process of sending abnormal cell components in the body, such as unfolded proteins, aging and damaged cells, and accumulated harmful substances, to lysosomes or vesicles for digestion, degradation, and recycling. The occurrence of autophagy provides cells with the energy and environment needed for survival, which is a self-protection mechanism for somatic cells (29). The lack or overactivation of autophagy will cause the destruction of the normal structure of cells and cell death. Therefore, the balance of the autophagy process plays an important role in maintaining the stability of the intracellular environment (30). The JAK/STAT signaling pathway is an important pathway involved in autophagy. Many studies have shown (31, 32) that by inhibiting the expression of JAK/STAT-related signaling pathways, cancer cell autophagy can be regulated, thereby inhibiting the proliferation of cancer cells and achieving the purpose of inhibiting the malignant progression of cancer. Simultaneously, it modulates cellular autophagy through JAK/STAT-associated signaling pathways, which is significantly critical in diseases such as rheumatoid arthritis and diabetes (33, 34).

#### 3.1.3 Control the inflammatory reaction

The JAK-STAT pathway is among the most expedited pathways in inflammation and serves as an essential pathway for inflammatory signaling (35). By inhibiting the activation of JAK/STAT-associated signaling pathways and modulating the release of various inflammatory mediators, it can ameliorate airway inflammation, influence skin barrier integrity, and restore the pathological condition of the gastric mucosa, thereby yielding a favorable therapeutic outcome for pulmonary disorders, inflammatory dermatoses, gastrointestinal ailments, and more (36–38).

#### 3.1.4 Inhibit oxidative stress reaction

Oxidative stress is a phenomenon of oxidation or imbalance in the body’s antioxidant system (39). When oxidation should be stimulated, the original redox dynamic balance in the cell is broken, resulting in the production of a large number of oxidation intermediate products, ROS, thus causing cell dysfunction and tissue organ damage. When the JAK1/STAT1 signaling pathway is inhibited, the oxidative stress response lobe is significantly inhibited, reducing brain tissue apoptosis, which reduces the apoptosis rate of neurons, plays a protective role in the brain, and is crucial in ischemia-reperfusion injury and many cardiovascular diseases (40).

### 3.2 Trend analysis of JAK1 clinical inhibitor research

(41–43) Since the first JAK inhibitor was discovered in the 1990s, many JAK1-targeting drugs have been approved globally. JAK1 has also become a prominent target for future drug development. To date, nine JAK1 drugs have been approved worldwide, which can be classified into two categories based on target selectivity: first-generation pan-JAK inhibitors (e.g., ruxolitinib, tofacitinib, and baricitinib) with lower selectivity and broader binding spectra, and second-generation agents with higher specificity. These first-generation inhibitors simultaneously bind to JAK1, JAK2, JAK3, TYK2, and other homologous targets, thereby blocking multiple downstream signaling pathways, thereby treating a variety of autoimmune diseases (41–43).

Second-generation inhibitors with high selectivity and specific binding to JAK1, including upadacitinib, abrocitinib, etc. For the first-generation pan-JAK inhibitors, safety has grown to be a major issue, though. For instance, improper inhibition of JAK2 might cause thrombocytopenia and anemia (44); improper inhibition of JAK3 might cause immunodeficiency and infection (45). Second-generation inhibitors have tremendously enhanced the safety of clinical drug use. They can specifically target particular JAK family members, so blocking particular disease-related signalling pathways without compromising the roles of other cytokines. Good clinical efficacy and safety of JAK1 inhibitors have been demonstrated by several studies (46–48). It is well known that JAK1 inhibitors help to treat atopic dermatitis and rheumatoid arthritis (49–51). Furthermore, the most recent development trend (52–54), and has great application possibilities is applying JAK1 inhibitors in gastrointestinal diseases, rheumatoid arthritis, and alopecia areata.

### 3.3 Clinical therapeutic effects of JAK1

JAK1 inhibitors have been demonstrated to possess multiple pharmacological effects and have become therapeutic agents for various diseases, including inflammatory bowel disease (55), rheumatoid arthritis (51), autoimmune diseases (56), and various cancers (57).

Inflammatory bowel disease (IBD) is a chronic inflammatory condition of the gastrointestinal tract, with Crohn’s disease (CD) and ulcerative colitis being its two primary forms (58). JAK1 inhibitors modulate the innate and adaptive immune responses involved in IBD by blocking JAK-mediated inflammatory pathways, thereby reducing chronic gastrointestinal inflammation (59). In the treatment of rheumatoid arthritis (RA), Janus kinase inhibitors (JAK inhibitors) are a new class of targeted therapies in addition to biologics (60). Studies have shown that JAK1 inhibitors can alleviate RA pain caused by both inflammatory and non-inflammatory mechanisms, and various cytokines directly or indirectly regulated by the JAK/STAT pathway play a crucial role in the various mechanisms mediating RA pain (61). Cytokines play a central role in the pathophysiology of autoimmune diseases. JAK inhibitors are increasingly being used in the treatment of autoimmune diseases such as rheumatoid arthritis, systemic sclerosis, alopecia areata, vitiligo, and systemic lupus erythematosus (56, 62, 63). Currently, JAK1 inhibitors are being extensively explored in oncology. Certain drugs, such as Ruxolitinib, have undergone clinical trials to assess their antitumour efficacy in various solid tumours. These include pancreatic cancer (64), breast cancer (65), metastatic lung cancer (66), non-small cell lung cancer (67), liver cancer (68), and myeloproliferative tumours (69), among others, with promising clinical outcomes. In summary, JAK1 shows great potential in the treatment of the aforementioned diseases.

## 4 Conclusion

This study used VOSviewer and Citespace to comprehensively analyze the research on JAK1 over the past 20 years and systematically reviewed the development of this field. The results showed that JAK1 mechanism research, clinical drug research and related disease spectrum were the main research areas during this period. Importantly, JAK1-related research has shifted over time from initial mechanistic studies to drug development in combination with clinical disease. It has been proven to be effective and safe in the treatment of Rheumatoid Arthritis, atopic dermatitis, alopecia areata and gastrointestinal diseases; this emerging trend is expected to become an important research focus in the future. As a pivotal target in autoimmune therapeutics, JAK1 inhibitors demonstrate global potential for transformative drug development. Future research should emphasize international collaboration across academic institutions by leveraging shared expertise in JAK1 mechanisms and clinical validation to address global healthcare needs.
